# Tart Cherry Juice and Seeds Affect Pro-Inflammatory Markers in Visceral Adipose Tissue of High-Fat Diet Obese Rats

**DOI:** 10.3390/molecules26051403

**Published:** 2021-03-05

**Authors:** Michele Moruzzi, Nora Klöting, Matthias Blüher, Ilenia Martinelli, Seyed Khosrow Tayebati, Maria Gabriella Gabrielli, Proshanta Roy, Maria Vittoria Micioni Di Bonaventura, Carlo Cifani, Giulio Lupidi, Francesco Amenta, Daniele Tomassoni

**Affiliations:** 1 School of Pharmacy, University of Camerino, 62032 Camerino, Italy; michele.moruzzi@unicam.it (M.M.); ilenia.martinlli@unicam.it (I.M.); khosrow.tayebati@unicam.it (S.K.T.); mariavittoria.micioni@unicam.it (M.V.M.D.B.); carlo.cifani@unicam.it (C.C.); giulio.lupidi@unicam.it (G.L.); francesco.amenta@unicam.it (F.A.); 2 Helmholtz Institute for Metabolic, Obesity and Vascular Research (HI-MAG) of the Helmholtz Zentrum München at the University of Leipzig and University Hospital Leipzig, 04103 Leipzig, Germany; nora.kloeting@helmholtz-muenchen.de (N.K.); matthias.bluher.medizin@uni-leipzig.de (M.B.); 3 Institute of Metabolic and Cardiovascular Diseases (I2MC), Institut National de la Santé et de la Recherche Médicale (INSERM), Université de Toulouse, 31432 Toulouse, France; 4 School of Biosciences and Veterinary Medicine, University of Camerino, 62032 Camerino, Italy; gabriella.gabrielli@unicam.it (M.G.G.); proshanta.roy@unicam.it (P.R.)

**Keywords:** obesity, visceral adipose tissue, inflammation, tart cherries

## Abstract

Background: Tart cherries (*Prunus cerasus* L.) are a rich source of anthocyanins. They are phytochemical flavonoids found in red and blue fruits, and vegetables that can reduce hyperlipidemia. Visceral Adipose Tissue (VAT) has emerged as a major player in driving obesity-related inflammatory response. Methods: This study has investigated the potential positive effects of tart cherries on rats with Diet-Induced Obesity (DIO). In particular, the inflammatory status in retroperitoneal (RPW) and perigonadal (PGW) adipose tissue were studied. Rats were fed *ad libitum* for 17 weeks with a hypercaloric diet with the supplementation of tart cherries seeds powder (DS) and seeds powder plus tart cherries juice containing 1mg of anthocyanins (DJS). In RPW and PGW, expression of CRP, IL-1 β, TNF-α, CCL2 and CD36, were measured by qRT-PCR, Western blot and immunohistochemistry techniques. Results: No differences in the weight of RPW and PGW animals were found between DS and DJS groups compared to DIO rats. However, an increase of inflammatory markers was observed in DIO group in comparison with control lean rats. A modulation of these markers was evident upon tart cherry supplementation. Conclusion: Study results suggest that tart cherry enriched-diet did not modify the accumulation of visceral fat, but it decreased inflammatory markers in both tissues. Therefore, this supplementation could be useful, in combination with healthy lifestyles, to modify adipose tissue cell metabolism limiting-obesity related organ damage.

## 1. Introduction

Obesity is a chronic and multifactorial disease, characterized by an increase in the white adipose tissue (WAT) that results in overstored fat, localized mainly in the abdominal region [1]. The adipose tissue (AT) is involved in different functions that include the storage and release of energy, thermal insulation, and internal organs protection. WAT is composed of adipocytes and a stroma vascular compartment containing pre-adipocytes, endothelial cells, fibroblasts, resident immune cells, blood vessels, and nerve terminals [2]. In obesity, the number of adipocytes looks to be tightly regulated and determined during childhood [3]. However, during obesity development, AT can expand by either hypertrophy or hyperplasia. Obesity is characterized by dysfunctional AT in which adipocytes initially become hypertrophic during periods of caloric excess. This state is followed by the recruitment of additional pre-adipocytes, which differentiate into mature adipocytes as compensatory protection against some of the adverse metabolic consequences of obesity [4]. Additionally, the AT possesses endocrine activity mediated by adipokines, a diverse set of signaling molecules that includes hormones, cytokines, acute phase reactants, and growth factors [5]. These molecules are essential in regulating tissues and organs such as the AT itself and the liver, the pancreas, and the brain metabolically active in maintaining energy homeostasis [6]. Mis-regulation of AT contributes to the development of insulin resistance and vascular complications of diabetes [7].

AT expansion in obesity is accompanied by inflammatory changes within AT contributing to chronic low-grade systemic inflammation that is characterized as slightly high elevated levels of circulating cytokines, chemokines, and acute phase reactants. In mice fed a high-fat diet, obesity is associated with the induction of several inflammatory pathways, constituting as much as 59% of total pathways that are differently regulated [8,9,10]. Expansion of AT depots during weight gain is accompanied by an infiltration of new inflammatory cells. Initially, the main one of them being are macrophages. Macrophages in AT of obese mice represent up to 60% of all cells, versus only 5–10% within AT of lean ones [9]. These pro-inflammatory cells are recruited in response to chemokines such as chemokine (C-C motif) ligand 2 (CCL2), also referred to as monocyte chemoattractant protein-1 (MCP-1), produced by hypertrophic adipocytes [11]. Studies in obese mice have demonstrated that most macrophages in obese AT are derived from circulating monocytes, although a small percentage appears to derive from a proliferation of resident tissue macrophages. These anti-inflammatory macrophages are believed to be responsible for maintaining tissue homeostasis. It remains unclear whether the derivation of AT macrophages is the same in human obesity [10]. During obesity, the increased levels of glucose, free fatty acids, reactive oxygen species (ROS), and hypoxia cause the activation of pro-inflammatory immune cytokines [12]. Oxidative stress results from an imbalance between production and inactivation of ROS, which contributes to cellular dysfunction [13].

Antioxidant dietary supplementations have beneficial effects on vascular dysfunction. Anthocyanins, as a group of flavonoids, are present in many fruits, vegetables, red wine, and grains [14,15]. Anthocyanins such as cyanidin-3-glucoside and pelargonidin-3-glucoside could be absorbed in their intact form into the gastrointestinal wall. After an extensive first-pass metabolism, their phenolic acid metabolites enter the systemic circulation in much higher concentrations than their parent compounds. These metabolites could be responsible for the health benefits associated with anthocyanins [16]. Anthocyanins were found in the bloodstream within minutes of consumption in humans, suggesting that they can be quickly absorbed from the stomach, confirmed in animal studies [16]. Anthocyanins were absorbed efficiently after in situ perfusions of the jejunum and ileum in rats. It was also demonstrated that anthocyanins could permeate cultured Caco-2 cell monolayers. Thus, the permeability of anthocyanins across the gastrointestinal mucosa was quite high, indicating that anthocyanins undergo extensive first-pass metabolism before entering the systemic circulation as metabolites [16].

Some studies showed that the higher consumption of anthocyanins was systematically associated with a lower risk of type-2 diabetes and cardiovascular disease onsets [17,18]. Anti-inflammatory compounds found in tart cherries (*Prunus cerasus* L.) seem to have strong antioxidant effects [19,20,21].

Anthocyanins have great biological activities and low toxicity in vivo. Therefore, many scientists are interested in the health benefits of anthocyanins. It is difficult to demonstrate the anti-inflammatory and anti-adipogenic effects in the cell lines [22]. Many studies in different rodent models of obesity demonstrated a reduction of the main inflammatory markers after tart cherry supplementation, for a review, see [22]. Recently, in male C57BL/6J tart cherry extract failed to reverse the effects of the high-fat diet (HFD) on body weight and glucose tolerance, but significantly reduced the leptin and IL-6 levels and increased the antioxidant capacity [23]. Despite some inconsistencies, different studies in humans support the anti-inflammatory effects of cherries in healthy conditions, in sportsmen, during pathological conditions such as diabetes, cardiovascular disease, cognitive decline, and obesity [24]. A randomized and crossover pilot study demonstrated a significant reduction in pro-inflammatory molecule CCL-2, a reducing trend for tumor necrosis factor alpha (TNF-α) and a significant difference, compared to placebo, in erythrocyte sedimentation rate after consumption of 100% tart cherry juice for four weeks in overweight individuals [25]. These data potentially support that the tart cherry may markedly reduce the risk of inflammation associated with many chronic diseases, including cerebrovascular disease, diabetes, and obesity.

Besides, physicochemical and chemical characterization of tart cherry seeds demonstrated that they are mainly composed of polyunsaturated fatty acids, including oleic and linoleic acid, while palmitic and stearic acid were dominant saturated fatty acids. The kernel oil contains high levels of unsaturated fatty acids, and saturated fatty acids account for less than 10% of total fatty acids; furthermore, the approximate content of mono- and polyunsaturated fatty acids are 50 and 40% of the total fatty acids, respectively [26]. The sour cherry kernel has gained attention due to its composition of bioactive compounds. It is important for the cosmetic, pharmaceutical, and food industries, where it is mainly used [26].

Based on the evidence of the presence of different chemical compounds in juice and seeds of tart cherries, the aim of this study was the evaluation of their possible anti-inflammatory effects on the perigonadal (PGW) and retroperitoneal (RPW) visceral adipose tissue (VAT) in a rat model of Diet-Induced Obesity (DIO). In rodents, as in human, visceral depots are thought to be associated with metabolic complications and appeared to increase the risk of diabetes, hyperlipidemia and cardiovascular disease [10,27].

DIO rats represent a good model to study obesity following a high-fat diet (HFD) and related inflammatory processes in VAT. Rats were fed with a high-fat diet, supplemented with tart cherry seeds powder alone (DS) or tart cherry seeds powder plus tart cherry juice (DJS). Markers of macrophage infiltration, such as CCL2, Cluster of Differentiation 36 (CD36), and inflammation markers, C-Reactive Protein (CRP), TNF-α and Interleukin-1 beta (IL-1 β) were evaluated by quantitative Real Time PCR (qRT-PCR), Western blot (WB), and immunohistochemical techniques (IHC).

## 2. Results

After the sacrifice, PGW and RPW were collected and weighted. After 17 weeks of a HFD, PGW and RPW were significantly increased in the different obese groups compared to the rats fed with a standard diet, used as a control (CHOW rats). Tart cherry supplementation did not modify the weight gain and the AT deposition (Table 1).

### 2.1. qRT-PCR

**Figure 1:**
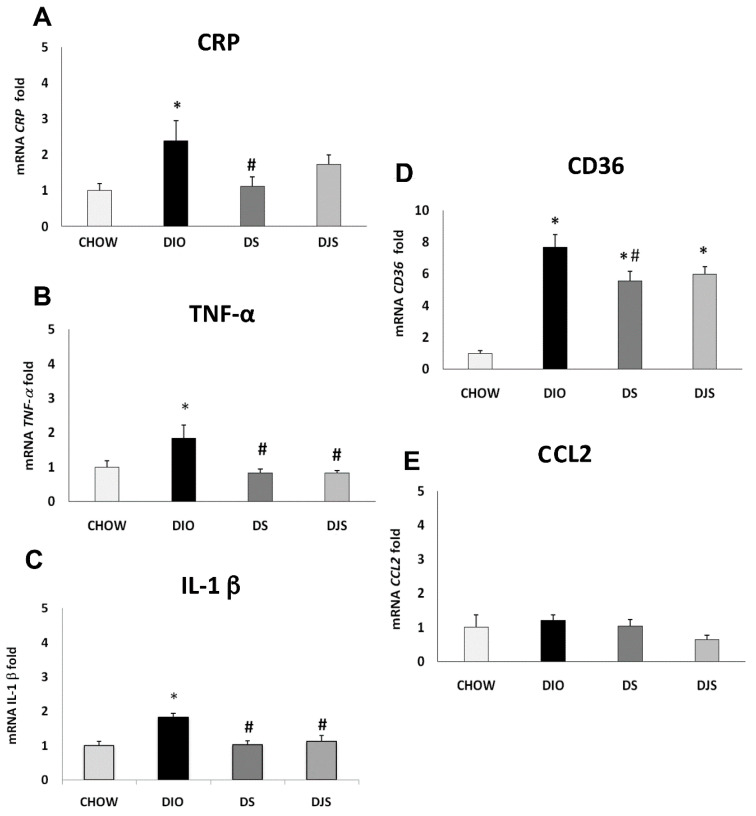
Gene expression analysis by Real-Time PCR in the perigonadal adipose tissue (PGW). (**A**) CRP, (**B**) TNF-α, (**C**) *IL-1 β*, (**D**) CD36, (**E**) CCL2. CHOW: standard diet; DIO: High-fat diet; DS. High-fat diet + Seeds; DJS: High-Fat diet + Juice and Seeds. Data are expressed as folds comparing DIO, DS, and DJS with CHOW rats used as control. Data are the mean ± SEM. * *p* < 0.05 vs. CHOW rats; # *p* < 0.05 vs. DIO rats.

**Figure 2:**
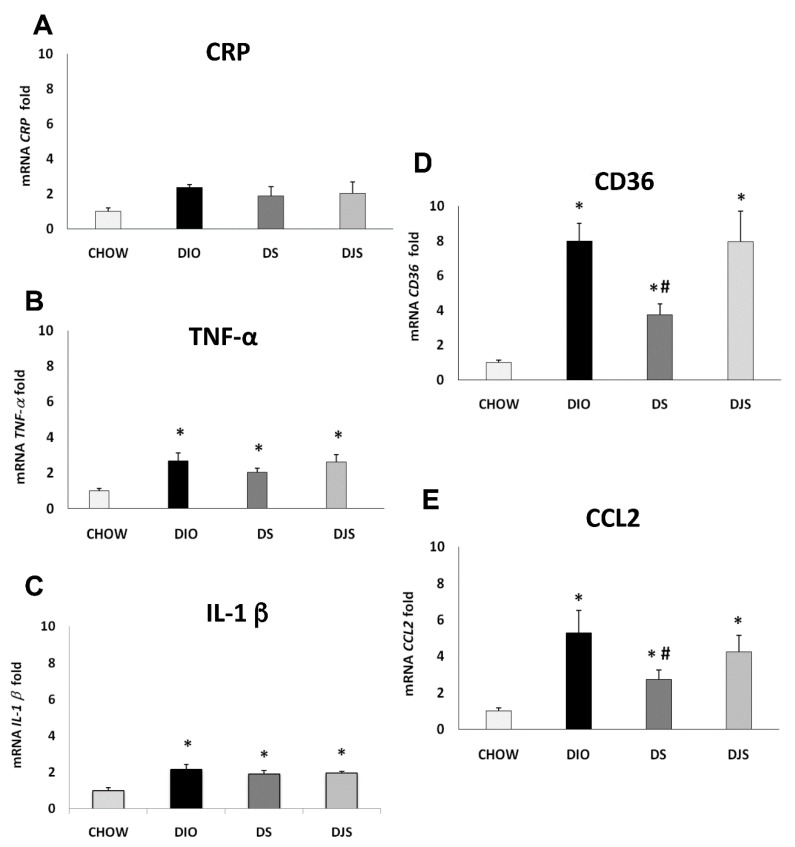
Gene expression analysis by Real-Time PCR in the retroperitoneal adipose tissue (RPW) (**A**) CRP, (**B**) TNF-α, (**C**) *IL-1 β*, (**D**) CD36, (**E**) CCL2. CHOW: standard diet; DIO: High-fat diet; DS. High-fat diet + Seeds; DJS: High-Fat diet + Juice and Seeds. Data are expressed as folds comparing DIO, DS, and DJS with CHOW rats used as control. Data are the mean ± SEM. * *p* < 0.05 vs. CHOW rats; # *p* < 0.05 vs. DIO rats.

Results of the qRT-PCR analysis for the gene expression of the CRP, TNF-α, IL-1 β, CD36, and CCL2 in PGW and RPW were summarized in Figure 1 and Figure 2. Results showed a significant generalized increase of pro-inflammatory cytokines in DIO rats compared to the CHOW group, especially for CRP and CD36 (Figure 1A,D, respectively) in PGW, and TNF-α, CCL2 and CD36 (Figure 2B,E,D, respectively) in RPW.

Remarkably, the reduced expression was found in particular for CRP and CD36 (Figure 1A,D, respectively) genes in PGW of the DS group compared to the DIO rats, suggesting that seed supplementation can reduce the inflammatory status induced by obesity. For these genes, no differences were found in the DJS group compared to DIO one. In contrast TNF-α and IL-1 β (Figure 1B,C, respectively) expressions were significantly down-regulated both in DS and DJS groups compared to DIO rats.

The qRT-PCR for the RPW (Figure 2) shows a significant increase of the TNF-α, *IL-1 β*, CD36 and CCL2 in DIO rats compared to CHOW rats (Figure 2B–E, respectively). In contrast, there was a significant reduction of CD36 and CCL2 in the DS group compared to DIO rats.

### 2.2. Western Blot Analysis

**Figure 3:**
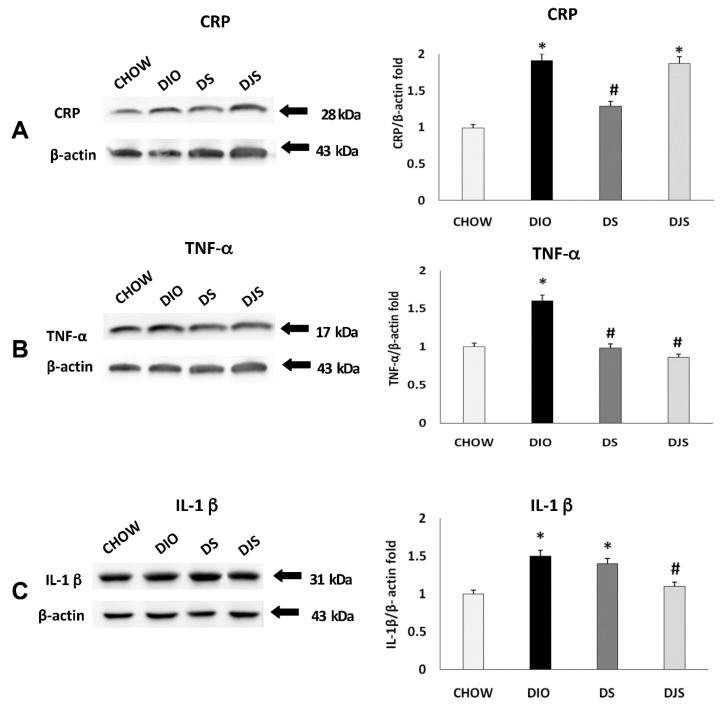
Immunochemical analysis of the PGW was processed with different antibodies: (**A**) anti-CRP, (**B**) anti-TNF-α, (**C**) anti-IL-1 β. CHOW: Standard diet; DIO: High-fat diet; DS. High-fat diet + Seeds; DJS: High-fat diet + Juice and Seeds. The densitometric analysis of bands is expressed as a ratio between the optical density of protein and reference protein (β-actin) where the value of the vehicle is set at 1. Data are the mean ± SEM. * *p* < 0.05 vs. CHOW rats; # *p* < 0.05 vs. DIO rats.

**Figure 4:**
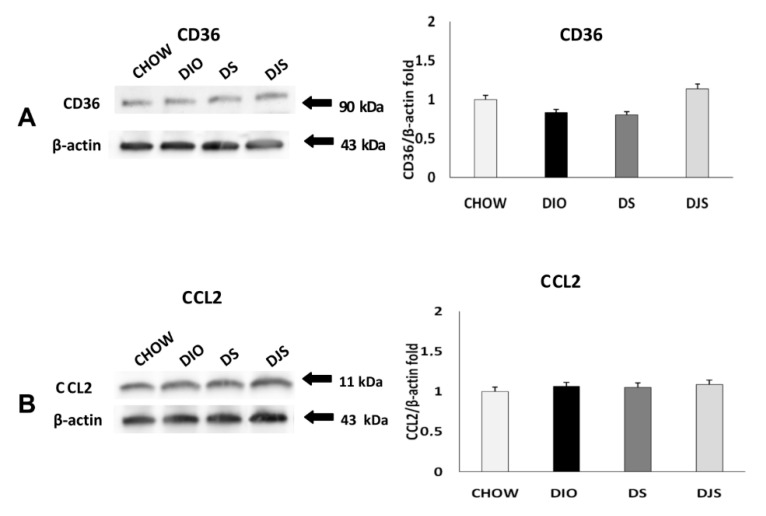
Immunochemical analysis of the PGW processed with different antibodies. (**A**) anti-CD36, (**B**) anti-CCL2 for CHOW: Standard diet; DIO: High-fat diet; DS: High-fat diet + Seeds; DJS: High-fat diet + Juice and Seeds. The densitometric analysis of bands is expressed as a ratio between the optical density of protein and reference protein (β-actin) where the value of the vehicle is set at 1. Data are the mean ± SEM.

The immunochemical analysis was performed for PGW and RPW samples. CRP, IL-1 β, TNF-α, CD36, and CCL2 were evaluated. This analysis revealed a pattern of bands at 28 kDa for CRP, 17 kDa for TNF-α, 31 kDa for IL-1 β, at 90 kDa for CD 36 and at 11 kDa for CCL2, approximately (Figure 3 and Figure 4).

Blotting assay for PGW showed an increased expression of inflammatory status in DIO rats, compared to CHOW group, with an increase of CRP, TNF-α, and IL-1 β (Figure 3). In the supplemented groups, different effects were observed: a reduction of the CRP and TNF-α expression were found in DS rats (Figure 3A,B), while IL-1 β and TNF-α decreased in the DJS group, as showed by the densitometric analysis of the bands (Figure 3B,C).

Regarding the monocyte molecule markers (Figure 4), there was no significant variation of expression for CD-36 (Figure 4A) and CCL2 (Figure 4B) among the groups.

**Figure 5:**
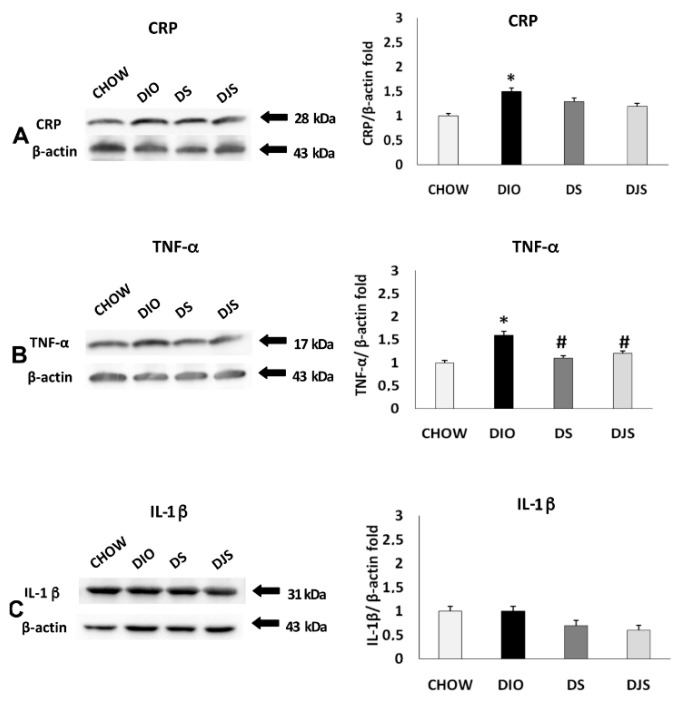
Immunochemical analysis of the RPW processed with different antibodies: (**A**) anti-CRP, (**B**) anti-TNF-α, (**C**) anti-IL-1 β. CHOW: Standard diet; DIO: High Fat diet; DS: High-Fat diet + Seeds; DJS: High-Fat diet + Juice and Seeds. The densitometric analysis of bands is expressed as a ratio between the optical density of protein and reference protein (β-actin) where the value of the vehicle is set at 1. Data are the mean ± SEM. * *p* < 0.05 vs. CHOW rats; # *p* < 0.05 vs. DIO rats.

Otherwise, immunochemical analysis for inflammatory markers in RPW (Figure 5) shows a divergent result than PGW. In this case, the TNF-α significantly decreased in both treated groups (DS, DJS) compared to DIO rats (Figure 5B). However, no difference was evident for IL-1 β in all groups (Figure 5C).

**Figure 6:**
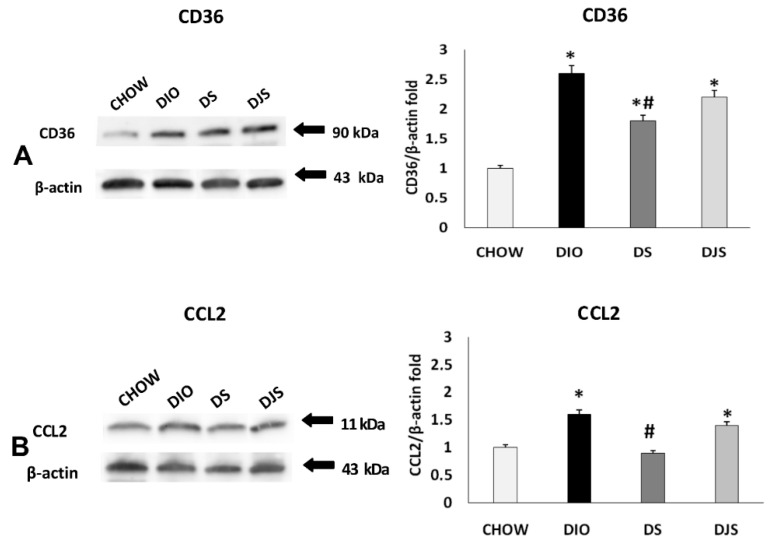
Immunochemical analysis of the RPW was processed with different antibodies (**A**) anti-CD36, (**B**) anti-CCL2. CHOW: Standard diet; DIO: High-fat diet; DS: High-fat diet + Seeds; DJS: High-fat diet + Juice and Seeds. The densitometric analysis of bands is expressed as a ratio between the optical density of protein and reference protein (β-actin) where the value of the vehicle is set at 1. Data are the mean ± SEM. * *p* < 0.05 vs. CHOW rats; # *p* < 0.05 vs. DIO rats.

WB analyses regarding the RPW tissue for the macrophage infiltration markers revealed an increase of CCL2 and CD36 in the DIO groups, compared to CHOW rats (Figure 6A,B, respectively). Besides, there was a significant reduction of both markers expression in the DS compared to the DIO group.

### 2.3. Immunohistochemistry

**Figure 7:**
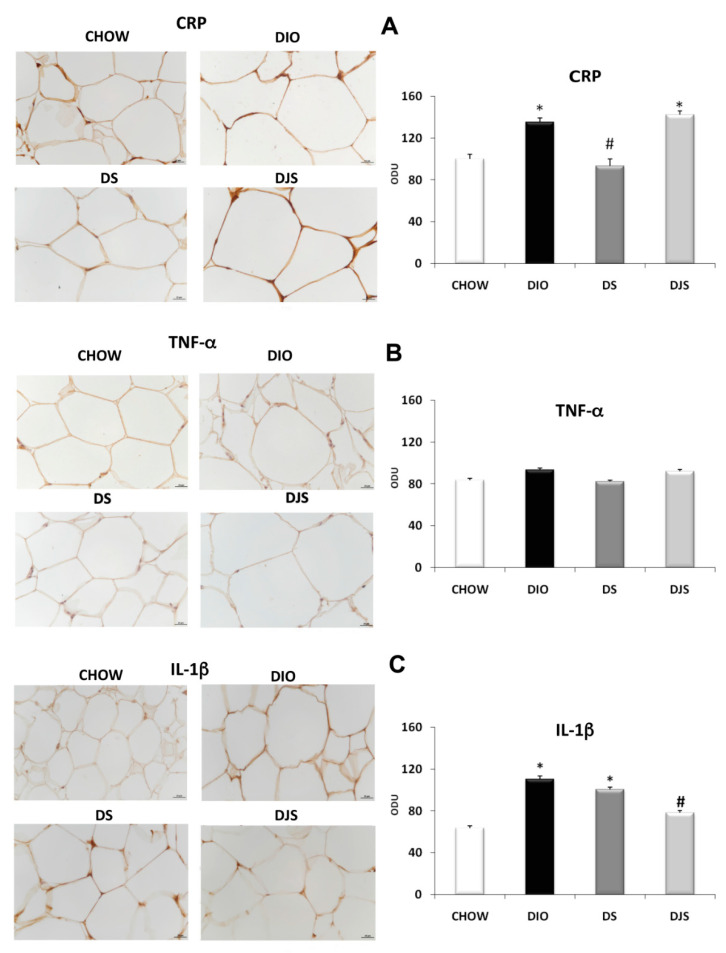
Immunohistochemical analysis of PGW processed with different antibodies: (**A**) anti-CRP, (**B**) anti-TNF-α and (**C**) anti-IL-1 β panel C. CHOW: standard diet; DIO: High-fat diet; DS. High-fat diet + Seeds; DJS: High-fat diet + Juice and Seeds. The densitometric analysis is expressed as an arbitrary optical density unit (ODU). Data are the mean ± SEM. * *p* < 0.05 vs. CHOW rats; # *p* < 0.05 vs. DIO rats.

IHC assay was performed in PGW tissues in CHOW, DIO, DS, and DJS rats as showed by representative pictures (Figure 7). A marked increase of immunoreactive density of the pro-inflammatory CRP and IL-1 β was evident in DIO rats compared with CHOW rats (Figure 7A,C, respectively). In addition, a significant reduction was found in the expression of CRP in DS and of IL-1 β in DJS groups with respect to DIO (Figure 7A,C, respectively). No change was observed in the TNF-α immunoreactivity (Figure 7B).

**Figure 8:**
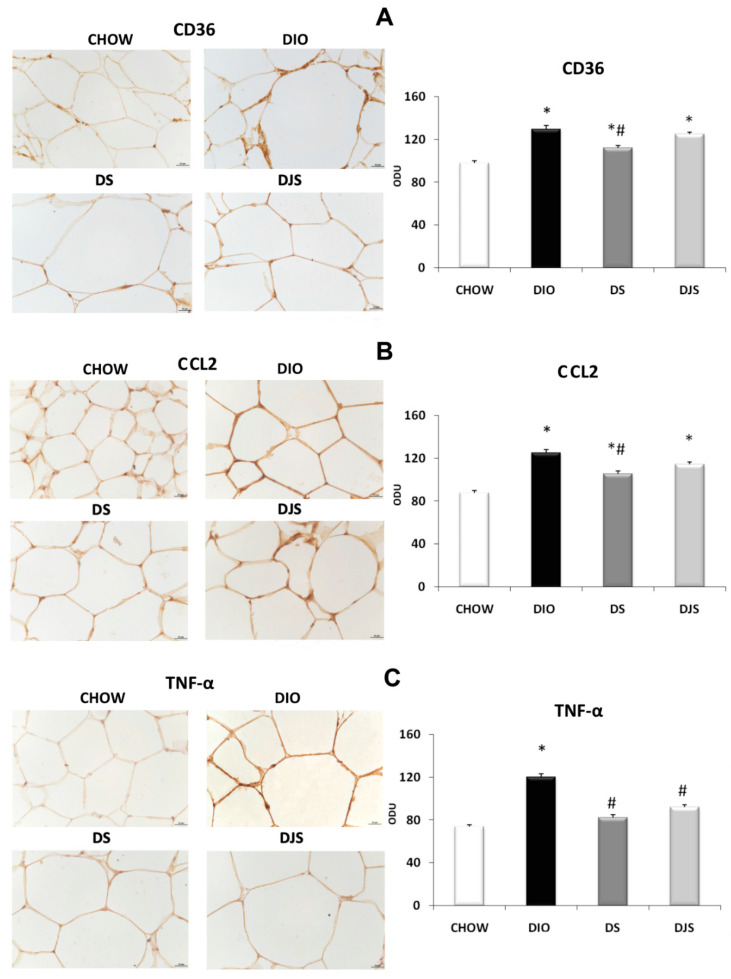
Immunohistochemical analysis of RPW processed with different antibodies: (**A**) anti-CD36, (**B**) anti-CCL2 and (**C**) anti-TNF-α. CHOW: Standard diet; DIO: High-Fat diet; DS: High-Fat diet + Seeds; DJS: High-Fat diet + Juice and Seeds. The densitometric analysis is expressed as an arbitrary optical density unit (ODU). Data are the mean ± SEM. * p < 0.05 vs. CHOW rats; *# p < 0.05* vs. DIO rats.

In RPW, a higher expression of CD36 was revealed in the DIO group than in the CHOW one (Figure 8A). A marked decrease in CD36 expression was observed in the DS group (Figure 8A). A similar pattern was also observed for CCL2 that increased its expression around adipocytes in the RPW of DIO rats. This immunoreaction was weak in the DS group and less extensive in the DJS one (Figure 8B). In RPW, a higher expression of TNF-α was revealed in the DIO group than in the CHOW one (Figure 8C). A decrease of TNF-α expression was showed in DS and DJS groups compared to DIO rats (Figure 8C).

## 3. Discussion

Obesity is considered a medical challenge because it is associated with chronic disease development. Actually high body mass index (BMI) or the increase of waist circumference is also correlated with the development of cardiovascular risk factors such as hypertension, dyslipidemia, insulin resistance, and diabetes mellitus [28,29,30,31]. In a recent meta-analysis, including 2.88 million individuals, all obesity grades combined were associated with a significant increase in mortality rate [32]. In obesity, the increased levels of glucose, free fatty acids, reactive oxygen species, and hypoxia cause the activation of pro-inflammatory immune cells [33]. CCL-2 is a chemotactic factor that promotes the recruitment of macrophages and monocytes in the site of inflammation. It is secreted by adipocytes during the development of obesity and is involved in the infiltration of monocytes, which differentiate to become macrophages of AT. Macrophages will secrete additional MCP-1 for further recruitment of inflammatory cells [34]. As fat mass expands in the obese, larger adipocytes become distant from the vasculature, come in to being ischemic areas [35]. Antioxidants, used as dietary supplementation, have beneficial effects on vascular dysfunction. Supporting the efficiency of such nutritional approaches, the positive effects of micronutrients found in natural products for controlling the pathogenesis of chronic diseases were highlighted [36].

Anthocyanins are the main subclass of phytochemical flavonoids that are primarily found in red-, blue-, and purple-pigmented fruits and vegetables. Several studies have suggested that anthocyanin-rich botanical extracts can modify lipid metabolism in vitro and can reduce hyperlipidemia in vivo [37,38,39,40,41]. These researches looked at how well tart cherries (*Prunus cerasus* L.) blocked lipid peroxidation or prevented cell membrane damage. All these compounds, including the base structure cyanidin (without sugar attached), and the three major sugar-added compounds, blocked this process and showed a similar effect. The high effects were obtained by cyanidin, meaning that the effectiveness of all its sugar products (anthocyanins) is probably due to the cyanidin “foundation”. Sugar products (glycosides) are formed by adding hydrogen or sugars, and it seems that the addition of small moieties (chemical groups) leads to the strongest antioxidant effect [24,42,43]. Furthermore, all tart cherry compounds matched standard commercial antioxidants in terms of antioxidant power [24,25]. Some randomized and crossover pilot studies had already confirmed the antioxidant effects of tart cherries (both as extract and as juice) in rats as well as in overweight or obese human subjects [25,44,45]. In addition, other studies have also demonstrated that incorporating bioactive food components in the diet exerts multiple beneficial health effects countering inflammation, obesity, and other metabolic disorders [46,47].

The results of the present study demonstrate that the inflammatory and macrophage infiltration markers showed a distinct pattern in the PGW and RPW as a consequence of the tart cherry supplementations. In fact, an anti-inflammatory effect of tart cherry supplementation can be deduced by all the techniques used mainly in the PGW while a reduction of macrophage infiltration markers was observed clearly only in RPW, in particular by the tart cherry seeds supplementation.

The seeds plus juice supplementation has been investigated to evaluate a possible improvement of the tart cherries juice performance that was previously investigated alone [41]. Moreover, the two supplementations were chosen also due to their active principles (flavonoids and fatty acids) that may be effective in a different way in the AT inflammatory process. Recently, it has been described that daily tart cherry juice consumption may counter the neuroinflammation in the brain [48].

Our rat model has shown a significant increase in body weight, according to the typical obese phenotype. In particular, the VAT weights from CHOW and DIO animals were significantly different, as previously demonstrated [48]. Although tart cherry seeds and juice did not affect body weight gain in DIO rats, both the systolic blood pressure and glycemia values were reduced, as previously reported [48]. Moreover, tart cherry supplementations did not prevent the fat accumulation of VAT, induced by an HFD ad libitum (45%), indicating no effect for what concerns the hypertrophy of adipocytes during obesity.

The effects of anthocyanins supplementation on the body weight remain controversial [37,39]. However, data confirmed that anthocyanins are health-promoting bioactive compounds [40]. Several studies have shown that phytocompounds found in fruits and vegetables have beneficial effects like prevention of cancer, cardiovascular diseases, and obesity [38].

Our data agree with other studies in which oral anthocyanin treatment did not preserve the rats from diet-induced weight gain [23,49,50]. Other investigations demonstrated that although tart cherry supplementation did not reduce the body weight in obese Zucker rats [51], it reduced inflammatory markers expression in VAT [51] and altered abdominal obesity and inflammation in obesity-prone rats fed HFD [52].

Increased AT mass during obesity increased the circulating levels of different adipokines [53,54]. The levels of TNFα increased proportionally to adiposity and insulin resistance. IL-1, one of the major inflammatory mediators, induced the expression of other inflammatory lymphokines, such as IL-6 [55].

Our data regarding the profile of cytokine expression in RPW and PGW confirm that pro-inflammatory cytokine genes are over-expressed in animals fed with HFD compared to controls, and the anti-inflammatory ability of the tart cherries. In particular, TNF-α and IL-1 β levels were reduced in DS and in the DJS groups, suggesting the possible effectiveness of both juice and seeds against the pro-inflammatory environment.

Our results regarding the infiltration macrophage markers, CD36 and CCL2, showed in DIO rats an increased expression in RPW, suggesting an inflammatory pattern unlike that in two anatomical compartments of VAT as a possible consequence of different fat deposition [56]. qRT-PCR, WB and IHC results showed a reduction of macrophage infiltration in treated rats, especially in the DS group, leading to the similar levels of CHOW groups in particular for CD36 expression. In contrast, in the DJS group, CD36 is expressed similarly to the DIO group, showing the weak effect of this kind of supplementations on macrophage infiltration. The main components of tart cherries seeds are oleic and linoleic acids, which have shown endothelium protection [57]. These compounds could partially justify the anti-inflammatory effects observed in the VAT of DIO rats. An enriched-diet in oleic acid may have a beneficial effect on type 2 diabetes and ultimately reverse the negative effects of inflammatory cytokines observed in obesity and non-insulin-dependent diabetes mellitus [58]. However, the anti-inflammatory activity of linoleic and oleic acids in the AT is quite controversial. In fact, a diet wealthy in polyunsaturated fatty acids, although modified the levels of adiponectin and fatty acid receptor proteins, did not affect other metabolic and inflammatory mediators in AT that were unchanged in HFD mice and rats [59,60]. Anyway, our data evidence the effects of tart cherry seeds containing mainly oleic and linoleic acid in reducing the inflammatory markers expressions and macrophage infiltration in VAT. Therefore, it is necessary to perform further studies to better clarify the role of these fatty acids in adipose tissue metabolism.

Based on much evidence, VAT not only acts as energy storage but also plays an important endocrine function. Adipokines, produced by AT, and secreted into the bloodstream during physiological conditions or in obesity, pathologically change the gene expression and secretory pattern of such compounds, significantly elevating the levels of proinflammatory molecules such as CCL2, IL-6, and resistin while decreasing the production of anti-inflammatory adiponectin and IL-10. These alterations contribute to the development of VAT inflammation, insulin resistance, and other symptoms of obesity.

The anti-inflammatory effects, induced particularly by tart cherry seeds, were not related to the body and VAT weight modulations. The changes of glycemia levels, the decrease of oxidative stress, and the downregulation of leptin in VAT [61,62], induced by tart cherry supplementation, could explain the anti-inflammatory effects observed in this study. Down regulation of leptin contributes to insulin resistance and show a correlation to the risk of metabolic syndrome, and leptin modulate immune reactions [63,64]. At the same time, the increase of adipokines consequent to HFD correlates with the degree of oxidative stress, with a reduction of superoxide dismutase activity [65]. As previously demonstrated, tart cherry supplementation can reduce the oxidative stress [61] and to modulate the levels of adipokines [62]. This could explain the anti-inflammatory effects at the level of VAT and, in particular, in the perigonadal depots.

In summary, although the tart cherry-enriched diet was not associated with significantly reduced body weight and abdominal fat, it is correlated with a reduction in VAT inflammation and macrophage infiltration that affects the levels of local adipokines. Tart cherry seeds and juice as a rich whole-food source of polyunsaturated fatty acid and anthocyanins may modify several key risk factors for type 2 diabetes. Further studies in subjects affected by obesity or metabolic syndrome could establish clinical benefits of tart cherry enriched diet.

## 4. Material and Methods

### 4.1. Animals, Tissue Processing and Treatment

The VAT samples used for this study were collected from the same male Wistar rats of 5 weeks of age (Charles River; n = 44; 250–275 g body weight) sacrificed as described in the study from Micioni Di Bonaventura et al. [48]. Male Wistar rats were used (n = 44) and divided into two groups: DIO rats (n = 36) and control rats (CHOW) (n = 8). The CHOW control rats were fed with the standard diet (4RF18, Mucedola, Settimo Milanese, Italy; 2.6 kcal/g).

At 7 weeks of age, DIO rats were fed with high fat (45%) diet ad libitum (D12451, Research Diets, Inc., New Brunswick, NJ, USA; 4.73 kcal/g. After 5 weeks (12 weeks of age), the obese phenotype starts to be developed. DIO group was further subdivided into two sub-groups:

- rats supplemented with 0.1 mg/g/day of tart cherry seeds powder (DS rats);
- rats supplemented with 0.1 mg/g/day of tart cherry seeds powder plus tart cherry juice, containing 1 mg of anthocyanins (DJS rats);

The HFD last for 17 weeks, until the 24 weeks of age of rats. The rats were anesthetized with carbon dioxide and sacrificed by decaitation. The RPW and PGW were taken based on the anatomical depots of visceral fat [27]. A portion of tissues was frozen in liquid nitrogen and stored at −80 °C for gene expression and biochemical analysis. Other portions were processed for IHC analysis using a fixative solution, containing 4% paraformaldehyde in 0.1 M phosphate buffer, pH 7.4, (PBS) at 25 °C. After fixation at room temperature, the samples were gradually dehydrated in ethanol and embedded in paraffin wax.

### 4.2. Preparation of Juice and Seed Powder from Tart Cherries

Tart cherries were kindly provided by Azienda Agricola Sigisas (Macerata, Italy). The seeds were removed from fresh cherries and the pulps were homogenized at room temperature, using a blender for 5 min and then an Ultra Turrax for 1 min. The homogenate was then centrifuged at 7000× *g* for 10 min and the supernatant (pulpe extract) was removed and stored at 4 °C. The precipitate (cherry residue) was further extracted under stirring in 96% ethanol for one night at room temperature. The solution was centrifuged at 10,000× *g* for 20 min and the supernatant (ethanol extract) was collected and evaporated using a Rotavapor (temperature 40 °C) to remove the ethanol from the content. The concentrated juice was added to the pulp extract and standardized; in this way, the rats could be given 1 mg of anthocyanins every day, added in the drinking water, for all weeks of treatment [48]. The total anthocyanin content was measured by the differential method [66].

The chemical, biological, functional, and technological properties of the tart cherry juice have been already described. There are some predominant compounds such as general cyanidin-3-*O*-(2′-glucosyl)-rutynoside in a range of 57% of the total amount of anthocyanins. The others compounds are cyanidin-3-*O*-glucoside, cyanidin-3-*O*-sophoroside, and cyanidin-3-*O*-rutinoside with, respectively, amount of 29%, 20%, and 19% of anthocyanins [26,67,68].

Previous studies have measured the total anthocyanins, total phenolic content and Trolox equivalent antioxidant capacity in the sour cherry juice [69,70,71].

The dried seeds, deprived of the shell, were ground and degreased, using petroleum ether as solvent, with two ultrasound-assisted extractions (UAE) for 20 min each. The ultrasound extraction, which increased the extraction efficiency of polyunsaturated fatty acids (PUFA), was carried out at lower temperature thus favoring the stability of heat-sensitive compounds, preserving the natural characteristics of the raw material [Italian patents: IT 102016000039003 (UA2016A002631)]. The fatty acid composition of defatted seeds obtained by the UAE procedure was characterized by Gas Chromatography/Mass Spectrometry (GS-MS) [72]. The analysis revealed that linoleic and oleic acids were the most abundant fatty acids (44.1% and 43.3%, respectively) of extracted defatted seeds, as previously characterized elsewhere [62]. In the diet of the supplemented DIO rats, 0.1 mg/g of defatted seed powder was added to 1 g of lard, placed in the cage in a special bowl. The CHOW and DIO control groups were not fed with tart cherries.

### 4.3. Quantitave Real Time PCR

Total RNA was extracted from RPW and PGW with the RNeasy Plus Mini Kit (Qiagen, Hilden, Germany) using the QiaCube (Qiagen) and reverse transcribed using the iScript cDNA synthesis kit (BioRad, Hercules, CA, USA). One microliter of the resulting cDNA products was used as a template for polymerase chain reaction qRT–PCR was performed using the IQ5 Multicolor Real-time PCR detection system (Bio-Rad), the RT^2^ SYBR Green qPCR Mastermix (Qiagen). QuantiTect Primer Assays specific for macrophage infiltration were used as specified in Table 2. Specific control primer: Glyceraldehyde 3-phosphate dehydrogenase (GAPDH) and 18S ribosomal RNA (18S). All samples were assayed in triplicates on the same plate. The PCR parameters were 10 min at 95 °C followed by 45 cycles of 95 °C for 15 s and 60 °C for 40 s. Measurement of GAPDH levels was used to normalize mRNA contents, and target gene levels were calculated by the 2^−ΔΔCt^ method.

### 4.4. Western Blot Analysis

Samples (0.1 ± 0.02 g) were homogenized in a Mixer Mill MM300 in lysis buffer containing protease inhibitor cocktail (Sigma Aldrich, Milan, Italy). After two centrifugations at 13,000 rpm (10 min at 4 °C), aliquots of the supernatant were used for protein assay against a standard of bovine serum albumin (BSA) fraction V using a PamReac AppliChem protein assay (Cat. 9048-46-8, PamReacAppliChem, Darmstadt, Germany). An equal amount of proteins (40 μg) were separated by sodium dodecyl sulfate polyacrylamide gel electrophoresis and transferred to nitrocellulose membrane by electroblotting in the Towbin buffer [73]. Transblotted membranes were incubated with polyclonal antibodies as detailed in Table 3. The specificity of the immune reaction was assessed using antibodies pre-adsorbed with peptides used for generating them. Blots were then washed in PBS + 0.5% of TritonX-100 (PBS-T) and incubated with the horseradish-peroxidase-linked (H + L) secondary antibody IgG (donkey anti-rabbit IgG Cat. No. 31460, goat anti-mouse IgG Cat. No. G-21040) a dilution of 1:5000 for 120 min at room temperature. Positive bands were visualized by an enhanced chemiluminescence system (Pierce^®^ ECL Western Blotting substrate, Cat. 32106, Thermofisher Scientific, Rockford, IL, USA). To normalize protein loadings, membranes were stripped and incubated with a mouse monoclonal anti β-actin antibody IGg2b (clone BA3R, Cat. No. MA5-15739-HRP, ThermoFisher Scientific., Rockford, IL, USA) at a dilution of 1:3000 in PBS-T overnight at 4° C. Band intensities were measured by densitometry with GeneSys (Syngene, Cambridge, UK).

### 4.5. Immunohistochemistry

Sections of the RPW and PGW, 10 µm thick, were cut using a microtome and collected on Superfrost plus slides. The VAT sections were exposed to different antibodies of inflammatory and macrophage markers, as detailed in Table 3. Antibodies were diluted in PBS-T 0.3%. Optimal antibody concentration was established in a series of preliminary experiments. Slides were incubated overnight at 4 °C with primary antibodies (Table 3). Non-specific binding of IgGs was prevented by incubating them with BSA 3% in PBS-T for 1 h. The product of the immune reaction was then revealed by incubating slides for 30 min at 25 °C with the specific biotinylated secondary IgGs (EnVision FLEX Mini Kit, Dako) of anti-goat, anti-mouse, and anti-rabbit diluted 1:200 in PBS-T. The immune reaction was then revealed with Diaminobenzidine (0.05% 3-3-diaminobenzidine dissolved in 0.1% H_2_O_2_) as a substrate. Slides were then washed, counterstained with hematoxylin, mounted on coverslips and viewed under a light microscope. Some sections were incubated with a non-immune serum instead of a primary antibody to assess the background of immunostaining. The intensity of immunostaining was assessed microdensitometrically only in the area of immunoreaction, using a NIS Element Software (Nikon, Florence, Italy), taking as 0 (white) the total absence of immunoreaction of the negative control and 256 (black) the highest value of immunoreactions [74].

### 4.6. Data Analysis

The averages of different parameters investigated were calculated from single animal data, and group means ± SEM were then derived from mean single animal values. The significance of the differences between the averages was analyzed by one-way analysis of variance (ANOVA) followed by the Newman–Keuls multiple range test correction, using Prisma software. The significance level was set for *p* < 0.05 to evaluate the difference between the studied groups.

## Figures and Tables

**Table 1:** Body weight (BW), perigonadal (PGW) and retroperitoneal (RPW) adipose tissue weight.

	BW	PGW	RPW
**CHOW**	557.2 ± 0.7	9.5 ± 1.1	8.8 ± 0.9
**DIO**	682.8 ± 17 **	19.6 ± 1.4 *	29.4 ± 3.8 *
**DS**	683.1 ± 29.7 **	16.9 ± 1.1 *	25.1 ± 11.8 *
**DJS**	689 ± 20.8 **	20.4±.2.5 *	29.4 ± 3.1 *

CHOW: Standard diet; DIO: High-fat diet; DS: High-fat diet + Seeds; DJS: High-fat diet + Juice and Seeds. Data are expressed in grams, as the mean ± SEM. * *p* < 0.05, ** *p* < 0.01 vs. CHOW rats.

**Table 2:** Probe and Primer used for qRT-PCR.

cDNA Probe/Prime	Assay ID	Company
CRP	Rn00567307_g1	Thermofisher
CD36	Hs00354519_m1	Thermofisher
CCL2	Rn00580555_m1	Thermofisher
IL-1 β	Rn00580432_m1	Thermofisher
TNF-α	Rn005620055_m1	Thermofisher
18S	Hs99999901_S1	Thermofisher

**Table 3:** The primary antibodies using for WB and immunohistochemistry IHC.

Primary Antibody	Clone	Host Animal	Company	Cat. No.	WB	IHC
CD36	*Polyclonal*	*Rabbit*	ThermoFisher	*PA5-27236*	1:1000	1:200
CRP	*Polyclonal*	*Rabbit*	ThermoFisher	*PA5-79070*	1:1000	1:100
TNF-α	*Monoclonal*	*Mouse*	Enquire Bioreagents	*712-MSM6-P1*	1:1000	1:200
CCL2	*Monoclonal*	*Mouse*	ThermoFisher	*MA5-17040*	1:500	1:200
IL-1 β	*Monoclonal*	*Mouse*	Santa Cruz Biotech	*SC32294*	1:500	1:200
β-actin	*Monoclonal*	*Mouse*	Thermofisher	* MA5-15739 *	1:1000	1:500

## Data Availability

The data presented in this study are available on request from the corresponding author.

## Sample Availability

Samples of the tart cherry fruits are available from the authors.
